# Arterial Stiffness as a New Predictor of Clinical Outcome in Patients with Polycythemia Vera

**DOI:** 10.3390/jcm13226811

**Published:** 2024-11-13

**Authors:** Olga Mulas, Alessandro Sestu, Alessandro Costa, Salvatore Chessa, Carla Vargiu, Ludovica Corda, Francesca Pittau, Giorgio La Nasa, Giovanni Caocci, Angelo Scuteri

**Affiliations:** 1 Hematology Unit, Businco Hospital, ARNAS Brotzu, 09134 Cagliari, Italy; 2 Department of Medical Sciences and Public Health, University of Cagliari, 09121 Cagliari, Italy; 3 Post Graduate Medical School of Internal Medicine, University of Cagliari, 09121 Cagliari, Italy; 4 Internal Medicine Unit, University Hospital “Duilio Casula”, AOU Cagliari, 09123 Cagliari, Italy

**Keywords:** polycythemia vera, arterial stiffness, pulse wave velocity, progression disease

## Abstract

**Background:** Thrombotic adverse events and disease progression are crucial in Polycythemia Vera (PV), as it stands as the leading cause of mortality. The pulse wave velocity (PWV) is a valuable indicator of arterial aging and often plays a significant independent role in contributing to cardiovascular adverse events (CV-AEs). The aim of this study was to examine the relationship between PWV and critical vascular function parameters, with the goal of identifying new predictive factors of vascular damage and exploring a potential connection with disease progression. **Methods:** Non-invasive aortic stiffness was assessed through carotid–femoral PWV measurement. PWV was measured using the SphygmoCor device. History of arterial or venous thrombosis (TAEs) or other CV-AEs was collected at baseline. PWV measurements were repeated at baseline, at 6 and at 12 months. **Results:** The study involved 28 PV patients aged 27 to 77 years, with 57.1% being male. Fourteen patients (50%) reported a high-risk thrombotic score at diagnosis, and 60.7% had at least one comorbidity. Multivariable regression models showed that hemoglobin levels were independently associated with PWV (β: 0.68, SE 0.24, *p* < 0.01). During the follow-up period (median duration 21.3 months, range 6–33), a total of 13 events were documented. Specifically, two patients exhibited a loss of response to treatment, four patients presented an increase in spleen diameters, three patients displayed an escalation of systemic symptoms, and three patients had a clear progression to secondary myelofibrosis. PWV (per 1 m/s: OR 1.70, 95% CI 1.00–2.91, *p* = 0.047) and leukocyte count (per 1 × 10^3^/μL: OR 1.47, 95% CI: 1.04–2.09, *p* = 0.043) were significant predictors of events, independently of waist circumference, blood pressure, treatment, and hematocrit. **Conclusions:** PWV has demonstrated its potential as an effective tool for monitoring PV patients. It stands as a clinical parameter that can predict the risk of progression in PV patients. Further investigation is essential to fully explore this potential. If successful, it could offer clinicians a valuable resource for effectively managing PV patients.

## 1. Introduction

Philadelphia-negative myeloproliferative neoplasms (MPNs) are chronic blood disorders that encompass Polycythemia Vera (PV), Essential Thrombocythemia (ET), and myelofibrosis (MF) [1]. These conditions result from the excessive production of blood cells, which can lead to thrombotic and hemorrhagic complications. They are characterized by the clonal proliferation of blood cells driven by specific mutations in *JAK2*, *CALR*, or *MPL* [2]. In the case of PV, most patients carry a *JAK2* mutation, with about 96% having a specific mutation in exon 14 (*JAK2* V617F) and an additional 3% showing mutations in exon 12 [3,4]. These oncoproteins cause constant activation of the JAK2 signaling pathway, resulting in the overgrowth of mature myeloid cells.

Polycythemia Vera is marked by an elevated red blood cell count [5]. This condition increases the likelihood of microvascular dysfunction, leading to symptoms such as migraine-like headaches, erythromelalgia, transient ischemic attacks, and coronary artery dysfunction. Thrombotic adverse events (TAEs) and the progression of the disease into secondary myelofibrosis (sMF) and acute myeloid leukemia (AML) significantly contribute to morbidity and mortality rates in PV [6].

Established risk factors in assessing PV patients’ thrombotic risk include advanced age and previous thrombotic history [7,8,9]. Individuals under the age of 60 who have no prior history of thrombosis are categorized as low risk, whereas those aged 60 or older or with a history of thrombotic events are considered to be at high risk [10,11]. Implementing these approaches has resulted in significant improvements in symptom control and cardiovascular morbidity. Furthermore, some treatments show potential for prolonging survival, including JAK inhibitors, interferons, and potentially curative allogeneic bone marrow transplantation (BMT) [12]. Despite this, understanding the risk factors for the progression of MPN and how they change over time is challenging.

Novel perspectives suggest that increased arterial (aortic) stiffness may be pivotal in finding tissue damage. Arterial stiffness can be easily measured in clinical settings by carotid–femoral pulse wave velocity (PWV). PWV is a good proxy of arterial aging [13], capturing the continuum of “healthy” as well as “early” arterial aging [14]. Higher PWV values (i.e., stiffer and older arteries) have been associated with cardiometabolic risk factors [15] as well as end-organ (heart, kidney, and brain) damage [16,17]. Additionally, PWV has been proven as an independent predictor of CV events [18]. To date, stiffer arteries have been associated with changes in endothelial function [19] and vascular wall remodeling [20,21]. Repeated thrombotic processes at the small artery level may cause microvascular rarefaction and, thus, greater PWV values [22]. Additionally, though crosstalk with vascular content has been less explored, increased blood viscosity may impact arterial stiffening [23].

Few studies consider the role of arterial stiffness in MPNs. An interesting study on patients with ET indicated that carotid artery stiffness advanced more rapidly in those with *JAK2* V617F-positive ET compared to control subjects [24]. The authors identified a link between the rise in *JAK2* mutation burden and the coronary calcium score [25]. On the other hand, the role of accelerated arterial aging, i.e., stiffer arteries as indicated by greater PWV values, in the risk of adverse events in PV has not been explored.

The aims of the present pilot study were to explore (1) whether the characteristics of the circulating blood impact PWV; and (2) whether arterial stiffness independently predicts the progression of disease in PV patients.

## 2. Materials and Methods

### 2.1. Study Population

Patients eligible for this cross-sectional study were recruited from those diagnosed with PV at the Hematology Department of Businco Hospital in Cagliari, Italy.

The inclusion criteria were as follows:

- Confirmed diagnosis of PV according to the 4th edition of the World Health Organization classification [26];
- Treatment with either phlebotomy or cytoreductive therapy with hydroxycarbamide.
- The exclusion criteria were as follows:
- Prior or current treatment with JAK2 inhibitors.

Patients received treatment to obtain Hct control as per international guidelines [12], with the goal of Hct reduction below 45%. Patients were followed as per local management practice based on clinical judgment.

Enrollment began in November 2021 and concluded in March 2023. The study was conducted in accordance with the Declaration of Helsinki and received approval from the local ethical committee. Patients provided informed consent under a protocol approved by the Cagliari Ethics Board (PROT. PG/2021/8545).

### 2.2. Clinical Assessment and Measured Parameters

Information collected at the enrollment included clinical data, such as constitutional symptoms, splenomegaly, systolic blood pressure (SBP), diastolic blood pressure (DBP), heart rate (HR), and body mass index (BMI); laboratory findings such as hemoglobin (Hb), hematocrit (HCT), platelet count (Plts), absolute neutrophil count (ANC), white blood cell (WBC) count, serum lactate dehydrogenase (LDH), erythropoietin level, bone marrow biopsy, and *JAK2* mutational status. In addition, the type of treatment administered, such as phlebotomies (Phl) or other cytoreductive therapy (CT), was collected. Hydroxycarbamide was the sole cytoreductive therapy administered.

History of arterial or venous thrombosis (TAEs) or other cardiovascular adverse events (CV-AEs) was collected at baseline. Arterial CV-AEs included arterial hypertension, heart arrhythmia, heart failure, aortic aneurysms, cardiomyopathy, and valvular heart disease. TAEs comprised angina, stroke, myocardial infarction, ischemic cerebrovascular events, thromboembolic disease or peripheral artery disease, and venous thrombosis. Other CV risk factors, including diabetes, dyslipidemia, increased BMI > 24.5 kg/m^2^, or severe renal insufficiency, were also considered. During follow-up, we considered events of worsening disease, resistance to treatment, CV-AEs, increased splenomegaly, anemia, exacerbated systemic symptoms, bone marrow fibrosis exceeding grade 2, and clear progression to myelofibrosis or acute myeloid leukemia.

### 2.3. Assessment of Pulse Wave Velocity (PWV)

Non-invasive aortic stiffness evaluation was performed with the carotid–femoral PWV assessment. PWV was measured using the validated SphygmoCor device (AtCor Medical, Cardiex; Sydney, Australia), whose validation and reproducibility have been previously published [27]. Pulse transit time was determined as the average of 10 consecutive beats. Transit time between carotid and femoral pressure waves was calculated using the foot-to-foot method. The distance traveled by the pulse wave was measured over the body surface, subtracting the carotid location–sternal notch distance from the sternal notch–femoral site distance. PWV measurements were repeated at baseline, at 6 and at 12 months. PWV was assessed as the difference between the initial and final PWV measurements. Delta PWV was then assessed as reported below:

Delta PWV = PWV_last evaluation_ − PWV_first evaluation_ (m/s)

### 2.4. Statistical Analysis

To compare measured variable levels between PV treatment groups, ANCOVA was performed to obtain age-adjusted means (±standard error).

The search for event predictors was assessed using a Cox Proportional Hazards model. The models were adjusted for the effects of waist circumference, blood pressure, treatment, hematocrit, WBCs, and PWV.

Backward elimination of nonsignificant covariates yielded the final more parsimonious model. Statistical analysis was performed with SAS on Demand for Academics—SAS Studio version 9.04 (SAS Institute Inc., Cary, NC, USA).

## 3. Results

### 3.1. Patients

Between November 2021 and March 2023, we assessed the eligibility of 45 patients diagnosed with PV, based on the WHO 2016 classification [1]. These individuals were under the care of the Hematology Unit at Businco Hospital in Cagliari, Italy. Out of the total, 17 patients were excluded from the study. The reasons for exclusion were as follows: 10 patients were misdiagnosed and did not have PV, 2 patients were lost during the follow-up period, and 5 patients did not complete the consent form. Consequently, 28 patients were enrolled in the study. The total cohort comprised 57.1% males, with a median age at study entry of 59 ± 12.2 years. The *JAK2* V617F mutation in exon 14 was detected in 96.4% of patients, while only one patient (3.6%) harbored a *JAK2* exon 12 mutation. Before the diagnosis of PV, a history of cardiovascular adverse events (CV-AEs) was reported in five patients (17.8%). Regarding comorbidities, 2 patients (7.1%) had diabetes mellitus, 10 patients (35.7%) had hypertension, and 4 patients (14.2%) had dyslipidemia.

Patients were then categorized into two groups based on treatment received: 12 patients (42.8%) received phlebotomy, and 16 (57.1%) underwent cytoreductive therapy. The only cytoreductive therapy was hydroxycarbamide with a median dose of 1000 mg (range 500–1000) with a mean duration of treatment of 4.1 ± 1.41 years. Given the significant age difference between the two groups (*p* = 0.001), patients’ characteristics are presented as age-adjusted means and standard errors unless otherwise specified (Table 1).

No statistically significant differences were observed between the treatment groups in terms of CV-AE history, metabolic and cardiovascular parameters, or hematological values (Table 1). Regarding arterial stiffness, assessed by PWV, mean values were comparable between the phlebotomy group and cytoreductive group (respectively, 10.0 ± 0.8 m/s vs. 10.2 ± 0.7 m/s, *p* = 0.93).

### 3.2. Arterial Stiffness and Blood Composition

As outlined in the Introduction, the current framework for understanding arterial stiffness, indexed as PWV, primarily emphasizes vessel wall structure and function. One aim of the present study was to recall the potential relevance of the rheological properties of blood on arterial vessels. Univariate regression analysis suggested that, together with age (r = 0.551, *p* < 0.001), SBP (r = 0.547, *p* < 0.001), waist circumference (r = 0.559, *p* < 0.001), and PWV at study entry significantly correlated with hemoglobin (r = 0.527, *p* < 0.001) and hematocrit (r = 0.340, *p* < 0.01), but not WBCs (r = −0.203, *p* = 0.30). Yearly changes in PWV between follow-up and study entry showed a significant correlation with yearly changes in WBCs over time (r = 0.424, *p* < 0.01). Multivariable regression models confirmed that hemoglobin levels were independently associated with PWV (β: 0.68, SE 0.24, *p* < 0.01). Similarly, changes in WBCs were independently associated with changes in PWV (per 1 × 10^3^/μL WBCs yearly change: β 0.45, SE 0.22, *p* < 0.05).

### 3.3. Arterial Stiffness as a Predictor of Worsening of Disease Status

During the follow-up period (median duration 21.3 months, range 6–33), a total of 13 events were documented. Specifically, one patient experienced a CV-AE, two patients exhibited a loss of response to treatment, four patients presented an increase in spleen diameters, three patients displayed an escalation of systemic symptoms, and three patients had a clear progression to secondary myelofibrosis. The characteristics of subjects based on event development are illustrated in Table 2. At study entry, the two groups of patients did not differ significantly, except for WBC count, which was higher in patients who developed an event (10.6 ± 4.7 vs. 7.0 ± 2.7, *p* = 0.016).

**Figure 1:**
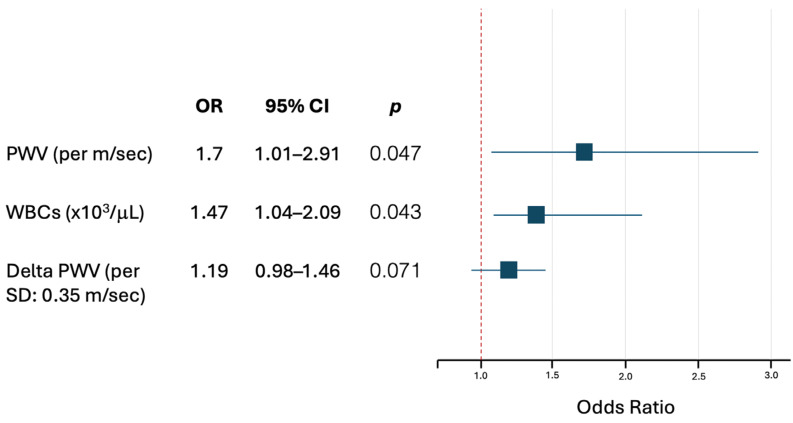
Logistic regression model considering WBCs and PWV as independent predictors of worsening events in PV patients. Abbreviations: PV, polycythemia vera; PWV, pulse wave velocity; SD, standard deviation; WBCs, white blood cells.

To assess the predictive role of arterial stiffness in disease progression among PV patients, multivariable logistic regression models were constructed. As illustrated in Figure 1, both PWV and WBCs were independent predictors of disease worsening. Notably, a 47% greater probability of having a new event was associated with a 1 × 10^3^/μL increase in total WBCs (OR 1.47, 95% CI: 1.04–2.09, *p* = 0.043). Stiffer arteries also predicted new events: an increase of 1 m/s in PWV was associated with a 70% greater probability of an event (OR 1.70, 95% CI 1.01–2.91, *p* = 0.047). Of note, a slight trend of borderline statistical significance was observable for changes in PWV over time (Delta PWV): a 0.35 m/s increase in PWV—corresponding to the median increase in the study population—was associated with 20% greater odds of event occurrence (OR 1.19, 95% CI 0.98–1.46, *p* = 0.071).

Finally, we explored whether changes in leukocyte count over time could serve as a predictor of events in our PV cohort. Changes in WBC count were incorporated into the predictive model either alongside or in place of baseline WBC count. In both cases, WBC changes over time did not emerge as independent predictors of events.

## 4. Discussion

Arterial stiffness, measured by PWV, results from vascular fibrosis and the degradation of elastic fibers in large arteries, leading to impaired arterial compliance. This phenomenon significantly contributes to the rise in systolic blood pressure associated with aging, thereby impacting overall prognosis [28]. Furthermore, arterial stiffness is an independent predictor of increased risk for stroke, coronary artery disease, and heart failure, irrespective of blood pressure levels [29]. Factors such as hypertension, high sodium intake, diabetes, dyslipidemia, obesity, and neurohormonal system activation can potentiate the aging process by influencing arterial stiffness [30].

A complex interplay of factors contributes to thrombotic events and increased mortality rates in individuals with PV, including advanced age, previous thrombotic events, elevated hematocrit, increased allele burden of the *JAK2* V617F driver mutation, and cardiovascular risk factors [31,32].

To the best of our knowledge, this study represents the first evaluation of PWV in a PV population. It investigated the potential of PWV as a marker to assess arterial stiffness and its relevance in evaluating signs of disease progression in PV patients. Notably, the PWV could be influenced by the components of blood cell counts [18,33,34]. We report a correlation between elevated hemoglobin and Hct levels and PWV in PV patients for the first time. Higher Hct levels imply increased erythropoietic activity, potentially leading to alterations in blood rheology that contribute to structural and functional changes in arterial wall properties [35]. Notably, these results are in accordance with previous findings reported by other groups. For instance, HCT levels significantly increased with PWV in 3225 adults (*p* = 0.001) [36], and a strong association between HCT and arterial compliance has been reported in a large cohort of 13.724 healthy adults or those with increasing cardiovascular risk profiles [37]. In PV management, cytoreduction predominantly aims to decrease the number of blood cells to normalize blood rheology [38]. However, cytoreductive therapy may contribute to reducing thrombotic risk independently of HCT reduction. We did not find a significant association between PWV and cytoreductive treatment, probably due to the limited number of patients analyzed. However, evidence showed that the positive effects of hydroxyurea on endothelial cells have already been reported, believed to occur by stimulating nitric oxide production and reducing leukocyte adhesion [39,40,41].

**Figure 2:**
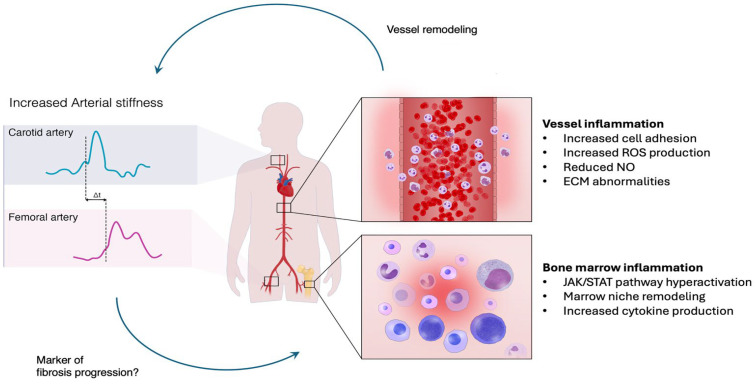
Relationship between PV-related bone marrow inflammation, vascular inflammation, and arterial stiffness. Abbreviations: ECM, extracellular matrix; NO, nitric oxide; ROS, reactive oxygen species.

Disease progression in MPNs involves intricate mechanisms that adversely affect prognosis. Key genetic factors include increased allele frequencies of oncogenic driver mutations and the acquisition of additional mutations in genes related to epigenetic regulation, transcriptional control, splicing, and tumor suppression, which confer a fitness advantage to malignant clones [42]. In addition to genetic factors, inflammatory processes significantly contribute to disease worsening. Cytokine-induced inflammation, arising from both malignant clones and non-hematopoietic bone marrow cells, promotes fibrosis and elevates cytokine levels that favor cancer cell growth. This inflammatory milieu also leads to abnormal expression of prothrombotic agents on endothelial cells, increasing the risk of thrombosis [43]. In our cohort, we found an interesting connection between high PWV and various negative events, including CVEs, lack of response to treatment, enlarged spleen, worsening systemic symptoms, and progression to secondary myelofibrosis. When combined, these events may lead to a worse prognosis for PV patients [44,45,46]. Indeed, symptoms could indicate the transition from a milder disease to a more severe one, like secondary myelofibrosis or acute myeloid leukemia. Furthermore, elevated leukocyte count correlated to increased risks of disease progression in our cohort. Prior studies have established the influence of leukocytes on inflammation and their role in advancing disease processes [47]. Inflammation has been recognized as a factor influencing arterial stiffness, independent of HCT control. Indeed, there are several associations between PWV and pro-inflammatory cytokines such as interleukin-6 (IL-6) and transforming growth factor-β (TGF-β) [48,49]. These pro-inflammatory mediators, widely expressed in MPNs downstream of *JAK2* V617F signaling, may thus play a role in determining arterial stiffness in PV patients (Figure 2) [50]. Through the NF-κB and SMAD pathways, these agents could trigger an inflammatory response in blood vessels, leading to increased cell adhesion, reactive oxygen species production, and changes in vascular compliance and extracellular matrix composition, ultimately resulting in vascular dysfunction and heightened arterial stiffness [51]. In MPN, evidence in the literature also suggests its association with bone marrow fibrosis development in pathogenic models of MPN, further supporting the validity of our study’s findings [52].

Notably, there seems to be a bidirectional relationship between the inflammatory process and hypertension. The mechanical stress caused by hypertension initiates a pro-inflammatory state, a recognized mechanism that contributes to arterial remodeling, endothelial activation, vasoconstriction, cellular proliferation, and the acquisition of a pro-inflammatory and prothrombotic pattern [53]. Conversely, increasing evidence implicates the immune system and the inflammatory response in the elevation of blood pressure. Cells from both the innate and adaptive immune systems, including monocytes, B cells, T cells, and natural killer cells, are all involved in hypertension. Indeed, immune cells migrate to target organs such as the arterial wall and end organs like the kidneys, heart, and brain, where the release of inflammatory cytokines contributes to vascular remodeling [54]. The renin–angiotensin system (RAS) also plays an important role in supporting both hypertension and inflammation [55]. This system is also involved in the development of certain myeloproliferative disorders, and its impact on blood vessels can be measured using PWV [55,56]. It has been shown that RAS inhibitors play a significant role in the management of PV and vascular stiffness [57,58]. Together, these interconnected mechanisms highlight the complex relationship between inflammation and hypertension, suggesting that targeting both processes may be essential for effective management.

Age is another critical, independent factor linked to survival in PV, and multiple factors contribute to arterial stiffening in response to aging, including interstitial matrix remodeling consequent to elastin fiber degradation, fibrotic deposition, inflammatory processes, and calcific alterations [8,59]. Furthermore, age-related arterial stiffening underlies the development of isolated systolic hypertension commonly observed in elderly patients [59].

In this field, PWV could be a simple tool to monitor subclinical progression. It could be used in combination with other factors, such as biomarkers for bone marrow fibrosis, the frequency of mutations in driver oncogenes, or other indicators of clonal evolution, to effectively manage PV in the future [60].

This study has several limitations that need to be considered. Firstly, the small sample size and single-center nature may have led to potentially biased results and may not fully represent the broader population of PV patients. Additionally, long-term follow-up data could provide a more precise and comprehensive understanding of the impact of PWV as a method for predicting and managing the risk of thrombotic complications in PV patients.

## 5. Conclusions

In conclusion, arterial stiffness—easily measurable as PWV—is associated with changes in hematocrit in patients with PV. Additionally, arterial stiffness represents an independent predictor of disease progression. Additional studies will clarify whether arterial stiffness is associated with or is causative of disease progression. These studies will also contribute to a better comprehension of the interplay between vascular wall and blood content on arterial aging, with a clear impact on hematological disorders management and prognosis.

## Figures and Tables

**Table 1:** Characteristics of the study population (age-adjusted means ± standard errors) or percentage. Statistical significance is indicated as *.

Characteristics	Phlebotomy (*n* = 12)	Cytoreductive Therapy (*n* = 16)	*p*
Sex male, *n* (%)	8 (66.6)	8 (50.0)	0.07 *
Time from diagnosis (mean, years)	4.6 ± 2.3	8.9 ± 3	
Age at study entry, mean years ± SE	52.2 ± 11.2	66.6 ± 9.4	0.001 *
Exon 14 *JAK2* V617F mutation, *n* (%)	11 (39.2)	16 (57.2)	
Exon 12 *JAK2* mutation, *n* (%)	1(3.6)	0 (0)	
Thrombotic risk score low, *n* (%)	7 (25)	5 (17.8)	
Thrombotic risk score high, *n* (%)	2 (7.1)	12 (42.8)	
CV-AEs before PV diagnosis, *n* (%)	2 (7.1)	3 (10.7)	0.32
Diabetes mellitus, *n* (%)	1 (3.5)	1 (3.5)	0.96
Dyslipidemia, *n* (%)	0 (0)	4 (14.2)	0.64
Hypertension, *n* (%)	2 (7.1)	8 (28.5)	0.08
WBCs × 10^3^/μL, mean ± SE	9.7 ± 1.3	7.8 ± 1.2	0.36
Hemoglobin g/dL, mean ± SE	14.3 ± 0.5	13.6 ± 0.5	0.37
Hematocrit, mean value ± SE	45.0 ± 1.5	43.5 ± 1.3	0.52
Platelets × 10^3^/μL, mean ± SE	408.9 ± 65.7	418.4 ± 60.1	0.92
BMI (Kg/m^2^), mean ± SE	24.8 ± 1.2	24.4 ± 1.1	0.93
Waist circumference, mean ± SE	92.8 ± 4.0	93.6 ± 3.7	0.90
SBP (mmHg), mean ± SE	129.8 ± 5.0	140.5 ± 5.4	0.20
DBP (mmHg), mean ± SE	82.3 ± 2.3	83.4 ± 2.5	0.76
HR (bpm), mean ± SE	78.8 ± 3.4	72.6 ± 3.1	0.29
eGFR (mL/min), mean ± SE	83.0 ± 4.4	88.6 ± 4.5	0.43
PWV (m/s), mean ± SE	10.0 ± 0.8	10.2 ± 0.7	0.93

Abbreviations: BMI, body mass index; CV-AEs, cardiovascular adverse events; DBP, diastolic blood pressure; eGFR, estimated glomerular filtration rate; HR, heart rate; PWV, pulse wave velocity; SBP, systolic blood pressure; SE, standard error; WBCs, white blood cells.

**Table 2:** Comparison of PV patients with or without disease progression at the time of study entry. Statistical significance is indicated as *.

	No Progression (*n* = 15)	Progression (*n* = 13)	*p*
Age at study entry, mean years ± SE	59.5 ± 11.4	60.2 ± 13.2	0.88
Sex male (%)	46.7	38.5	0.68
Phlebotomy (%)	46.2	46.7	0.97
Hypertension (%)	66.7	38.5	0.15
Diabetes mellitus (%)	6.7	7.7	0.92
Hemoglobin g/dL, mean ± SE	14.4 ± 1.8	13.4 ± 1.4	0.11
Hematocrit, mean value ± SE	45.1 ± 5.0	43.2 ± 3.7	0.28
WBCs × 10^3^/μL, mean ± SE	7.0 ± 2.7	10.6 ± 4.7	0.016 *
SBP (mmHg), mean ± SE	136.9 ± 21.6	132.3 ± 15.4	0.53
DBP (mmHg), mean ± SE	82.9 ± 10.0	82.7 ± 5.6	0.94
HR (bpm), mean ± SE	74.0 ± 9.3	77.7 ± 10.4	0.43
BMI (Kg/m^2^), mean ± SE	25.3 ± 3.6	23.8 ± 3.7	0.29
Waist circumference, mean ± SE	96.3 ± 11.7	89.8 ± 15.0	0.21
PWV (m/s), mean ± SE	10.0 ± 2.8	10.2 ± 2.8	0.86

Abbreviations: BMI, body mass index; CV-AEs, cardiovascular adverse events; DBP, diastolic blood pressure; eGFR, estimated glomerular filtration rate; HR, heart rate; PWV, pulse wave velocity; SBP, systolic blood pressure; SE, standard error; WBCs, white blood cells.

## Data Availability

Data are contained within the article.
